# Green exercise and kinesiophobia among Chinese adolescents: the mediating role of self-efficacy

**DOI:** 10.3389/fpsyg.2026.1743182

**Published:** 2026-01-28

**Authors:** Mingmin Kong, Yawei Ren, Tong Liu, Zhonggen Yin

**Affiliations:** 1 School of Sports Training, Chengdu Sport University, Chengdu, Sichuan, China; 2 College of Physical Education and Health Management, Chongqing University of Education, Chongqing, China

**Keywords:** activity avoidance, adolescents, green exercise, kinesiophobia, self-efficacy, social cognitive theory, somatic focus

## Abstract

**Objective:**

Kinesiophobia is a common psychological barrier to physical activity during adolescence and is characterized by fear-related attention to bodily sensations and avoidance of movement. This study examined whether green exercise is associated with lower kinesiophobia among adolescents and whether self-efficacy statistically mediates these associations, drawing on social cognitive theory.

**Methods:**

A cross-sectional questionnaire survey was conducted among 1,099 Chinese students in Grades 5–9 (aged 10–15 years); adolescents. Participants completed validated self-report measures of green exercise (5 items), self-efficacy (10 items), and kinesiophobia (13 items; somatic focus and activity avoidance). Descriptive statistics, multiple regression (controlling for gender, grade, and place of residence), structural equation modeling (SEM), and bias-corrected bootstrap tests (5,000 resamples) were used to evaluate the hypothesized relationships.

**Results:**

In multiple regression analyses, green exercise (*β* = −0.217; *β* = −0.289) and self-efficacy (*β* = −0.279; *β* = −0.266) were significant negative predictors of somatic focus and activity avoidance, respectively (all *p* < 0.001), with modest explained variance (*R*^2^ = 0.194 and 0.232). In SEM, green exercise was positively associated with self-efficacy (*β* = 0.351, *p* < 0.001) and showed significant direct associations with somatic focus (*β* = −0.251, *p* < 0.001) and activity avoidance (*β* = −0.315, *p* < 0.001). Self-efficacy was negatively associated with somatic focus (*β* = −0.308, *p* < 0.001) and activity avoidance (*β* = −0.299, *p* < 0.001). Bootstrap analyses supported partial indirect effects via self-efficacy for somatic focus (*β* = −0.108; 30.08% of total effect) and activity avoidance (*β* = −0.105; 25.00% of total effect).

**Conclusion:**

Findings indicate that greater participation in green exercise is associated with lower kinesiophobia in adolescents, partly through higher self-efficacy. The pattern is consistent with a social-cognitive mechanism linking outdoor physical activity to fear-related responses to movement, while causal inferences require longitudinal or experimental research.

## 1 Introduction

With the changes in modern society’s lifestyle, kinesiophobia has become a significant psychological barrier affecting individual health. Kinesiophobia refers to an excessive and irrational fear of physical activity or exercise due to the fear of potential harm or re-injury to the body (Kori et al., 1990). Studies have shown that kinesiophobia is often accompanied by strong anxiety and avoidance behaviors related to physical discomfort, poor physical ability, and social evaluation during exercise (Woolnough et al., 2022). This emotional response not only limits an individual’s participation in physical activity but may also lead to weight gain, physical health issues, and the further exacerbation of psychological disorders (Varallo et al., 2020). Kinesiophobia is particularly prominent among adolescents, affecting both their physical and mental health, while also hindering the establishment of a healthy lifestyle (Saulicz et al., 2016). Adolescents often face loneliness and anxiety when adapting to new environments, and social anxiety and low self-esteem may lead them to reduce their participation in physical activity, further exacerbating kinesiophobia (Rangul et al., 2024; Chen et al., 2023). Therefore, understanding the causes of kinesiophobia in adolescents and its intervention mechanisms is of great importance for improving their mental health and promoting a healthy lifestyle.

Green exercise refers to physical activities such as running, cycling, and hiking conducted in natural environments (Loureiro and Veloso, 2017). In recent years, green exercise has gradually been applied as an effective psychological health intervention to alleviate emotional issues such as psychological stress and anxiety (Li et al., 2022; Barton and Pretty, 2010). Compared to indoor exercise, green exercise combines the relaxation effects of natural landscapes with the physiological benefits of physical exercise, providing unique psychological health advantages for individuals (Wicks et al., 2022). Research has shown that green exercise significantly reduces anxiety, depression, and stress, improving individuals’ emotional stability and well-being (Carter et al., 2022). Additionally, the restorative effects of green exercise and its ability to enhance psychological resilience are facilitated through natural landscapes and social interactions (Hu et al., 2025). Although the positive impact of green exercise on mental health has been well established (Hu et al., 2025; Flowers et al., 2018), its role in reducing kinesiophobia remains unclear. In particular, how green exercise reduces kinesiophobia by enhancing individuals’ self-efficacy has not been systematically studied.

Self-efficacy refers to an individual’s belief in their ability to complete specific tasks, influencing their behavior choices, effort levels, and emotional regulation (Bandura, 1977). In exercise contexts, self-efficacy significantly affects individuals’ exercise behaviors (McAuley et al., 2011; Blom et al., 2021). Research indicates that higher self-efficacy helps individuals overcome negative emotions, such as fear and anxiety, during exercise, thus promoting continued physical activity (Zhu et al., 2024; Tang et al., 2022). Green exercise, by improving individuals’ physical health, increasing feelings of achievement, and boosting self-confidence, contributes to enhancing their self-efficacy (Reed et al., 2013; Glover and Polley, 2019). Therefore, self-efficacy may be the key psychological mechanism through which green exercise reduces kinesiophobia. Although studies have shown that self-efficacy plays an important role in exercise behaviors, the mechanism through which green exercise enhances self-efficacy to alleviate kinesiophobia has not been fully verified.

This study aims to explore how green exercise reduces kinesiophobia in adolescents by enhancing self-efficacy. Specifically, the study will address the following questions: (1) Can green exercise effectively reduce kinesiophobia? (2) Does self-efficacy mediate the relationship between green exercise and kinesiophobia? Through SEM and survey questionnaires, this study aims to reveal how green exercise alleviates kinesiophobia by enhancing self-efficacy. The findings will not only enrich the theoretical framework of green exercise in sport psychology but also provide empirical evidence for exercise interventions and psychological health promotion.

## 2 Literature review and hypotheses

### 2.1 Definition and current research on kinesiophobia

Kinesiophobia is a psychological disorder characterized by an individual’s persistent and excessive fear of and avoidance toward physical activity (Kori et al., 1990). Studies have found that kinesiophobia is often accompanied by intense anxiety and avoidance related to physical discomfort during exercise, poor physical abilities, and social evaluation during the exercise process (Mason et al., 2019; Levinson et al., 2013). This emotional response not only limits an individual’s participation in physical activity but may also lead to weight gain, physical health issues, and psychological disorders (Gabrys and Wontorczyk, 2023; Wingo et al., 2013). Kinesiophobia is particularly prevalent among adolescents, as research indicates that adolescents often face loneliness and anxiety when adapting to new environments, which may affect their exercise behavior. For instance, social anxiety during the initial school period may lead adolescents to reduce their physical activity participation (Wan et al., 2025; Jang and Park, 2024). From the perspective of social cognitive theory, an individual’s behavior is influenced by the interaction of cognitive, emotional, and social environmental factors (Bandura, 1991). The formation of kinesiophobia is closely related to an individual’s cognitive evaluation of their physical abilities and their concerns about social judgment. Although cognitive behavioral therapy (CBT) and exposure therapy have made some progress in treating kinesiophobia (Zhu et al., 2024; Yang et al., 2022; Lenouvel et al., 2023), these methods rely on long treatment periods and are influenced by individual psychological adaptability differences. Therefore, exploring new intervention methods, especially those that can naturally provide emotional restoration and psychological relaxation, such as green exercise, is an urgent research direction.

### 2.2 Psychological benefits and intervention mechanisms of green exercise

Green exercise refers to physical activities conducted in natural environments, such as running, cycling, and hiking (Loureiro and Veloso, 2017). It combines the restorative effects of natural environments with the physiological benefits of physical exercise, gradually becoming an effective psychological health intervention method (Han, 2021; Lahart et al., 2019). The psychological benefits of green exercise are primarily reflected in the following mechanisms: Psychological restoration mechanism, Attention Restoration Theory suggests that exposure to natural landscapes helps individuals recover attention resources depleted by prolonged stress and cognitive load, thereby improving mood and emotional states (Kaplan and Kaplan, 1989). Studies have shown that green exercise significantly reduces negative emotions such as anxiety and depression, promoting psychological recovery (Das and Gailey, 2022; Firnhaber et al., 2025; Coventry et al., 2021). Physiological activation mechanism, green exercise enhances physical fitness and improves cardiovascular health, and while improving physical health, it also contributes to increased overall well-being (Christie et al., 2015; Twohig-Bennett and Jones, 2018). Social support mechanism, green exercise typically has a strong social aspect, as individuals often interact with others while engaging in outdoor activities, thereby enhancing feelings of social support and belonging, which helps alleviate loneliness and anxiety (Song and Lin, 2022). These mechanisms work together, making green exercise an effective intervention for alleviating mental health issues such as anxiety, stress, and depression. However, despite the evidence confirming the positive impact of green exercise on mental health, especially in reducing anxiety and depression (Wicks et al., 2022; Gladwell et al., 2013), its role in reducing kinesiophobia remains unclear. In particular, how green exercise reduces kinesiophobia by enhancing self-efficacy remains an unexplored research gap.

### 2.3 The role of self-efficacy in exercise behavior

Self-efficacy is an individual’s belief in their ability to complete tasks in specific situations (Bandura, 1977). In the context of exercise, self-efficacy has been proven to be a key psychological factor affecting individual exercise participation. Individuals with higher self-efficacy are typically able to overcome negative emotions during exercise, such as fear and anxiety, and persist in exercising (Wu et al., 2025; Tang et al., 2022). In the context of green exercise, the enhancement of self-efficacy occurs mainly through two pathways: first, the restorative effects provided by the natural environment and the sense of achievement during exercise enhance the individual’s confidence; second, green exercise often involves social interaction, which strengthens the sense of social support, thereby promoting an increase in self-efficacy. Studies show that an increase in self-efficacy helps reduce kinesiophobia and increases motivation to engage in exercise (Sheng et al., 2025; McAuley et al., 2011; Reide et al., 2023). Therefore, self-efficacy may play an important mediating role in the green exercise intervention for reducing kinesiophobia.

### 2.4 Mediating mechanism of green exercise in reducing kinesiophobia through self-efficacy

According to social cognitive theory, an individual’s behavior is influenced by environmental, cognitive, and emotional factors (Bandura, 1991). Self-efficacy, as an important variable in this process, determines an individual’s behavioral responses when facing stress and challenges. In the exercise context, higher self-efficacy significantly reduces kinesiophobia and enhances the individual’s willingness to engage in exercise (Lavín-Pérez et al., 2024; Klompstra et al., 2018). Thus, green exercise may have a unique psychological mechanism, playing a role in enhancing self-efficacy and alleviating kinesiophobia. First, green exercise provides a low-pressure, restorative environment, allowing individuals to exercise without feeling excessive anxiety (Schmid et al., 2021); second, through social interactions and the support of the natural environment, green exercise strengthens an individual’s self-confidence and psychological resilience (Mnich et al., 2019; Rogerson et al., 2020). The enhancement of self-efficacy makes it easier for individuals to overcome fears and discomfort during exercise, thus increasing their interest and participation in physical activity (Wu et al., 2025; Tang et al., 2022). Therefore, green exercise may play a mediating role in reducing kinesiophobia by enhancing self-efficacy.

### 2.5 Theoretical model

Based on the aforementioned literature review, this study proposes the following hypotheses:

*H1*: green exercise has a negative impact on adolescents’ somatic focus.

*H2*: green exercise has a negative impact on adolescents’ activity avoidance.

*H3*: self-efficacy mediates the relationship between green exercise and adolescents’ somatic focus.

*H4*: self-efficacy mediates the relationship between green exercise and adolescents’ activity avoidance.

**Figure 1:**
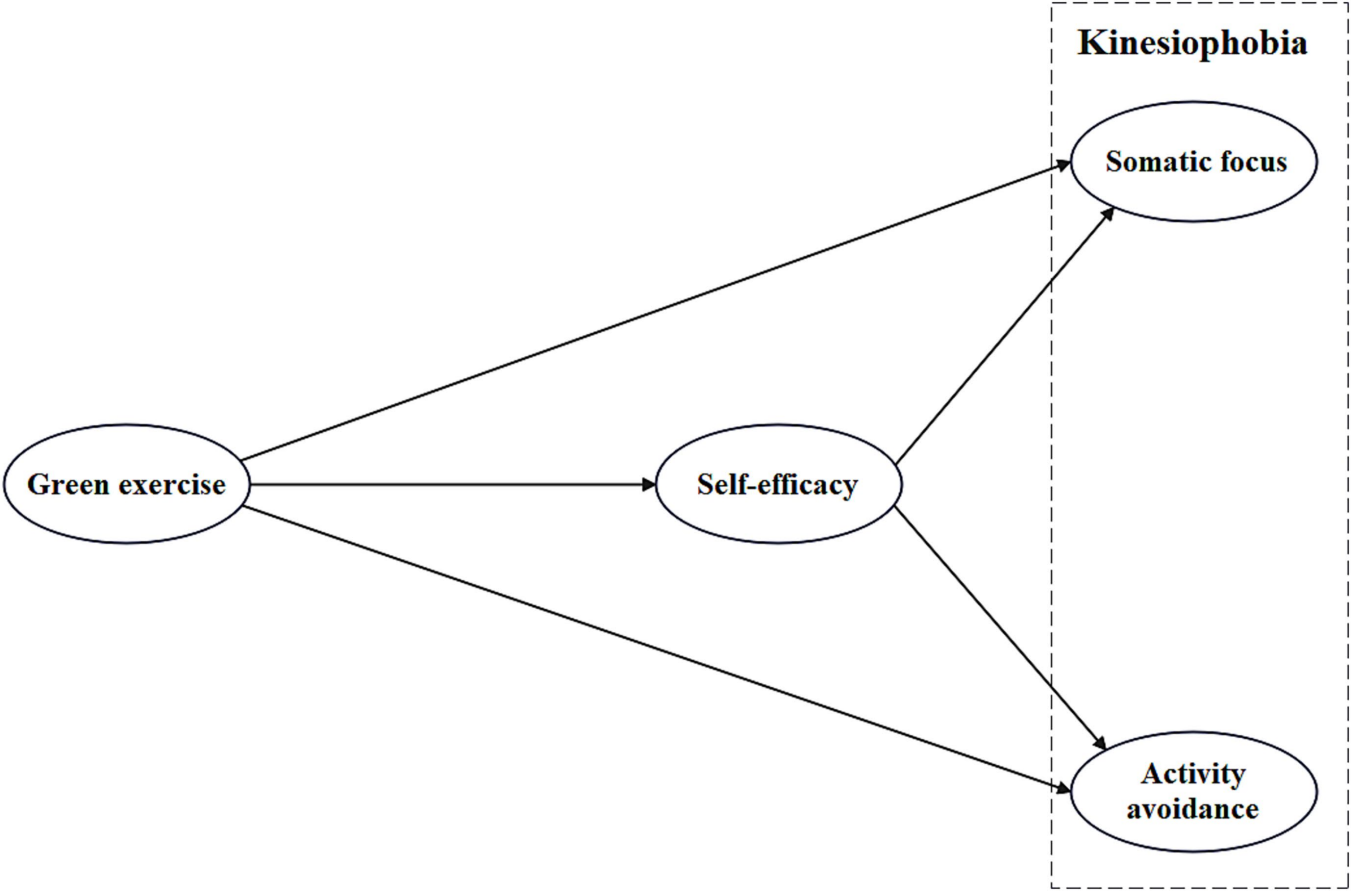
The theoretical model of the effect of green exercise on kinesiophobia through self-efficacy.

With the above hypotheses as the foundation, a theoretical model has been developed, as shown in Figure 1.

The model hypothesizes that green exercise indirectly influences adolescents’ somatic focus and activity avoidance through self-efficacy. In this process, self-efficacy is expected to play a significant mediating role, facilitating the negative effects of green exercise on adolescents’ somatic focus and activity avoidance. By integrating these variables, this study’s theoretical framework provides a systematic perspective for understanding the multi-dimensional mechanisms through which green exercise influences adolescents’ mental health. Moreover, this framework offers theoretical support and practical guidance for developing psychological health promotion strategies based on green exercise, helping schools enhance adolescents’ participation in green exercise and self-efficacy. This, in turn, can effectively improve mental health, reduce the negative impacts of Somatic focus and activity avoidance, and strengthen psychological resilience.

## 3 Materials and methods

### 3.1 Research participants and data

To ensure the representativeness and scientific validity of the sample, this study employed a stratified random sampling method to select school students from regions including Sichuan, Chongqing, and Guizhou. The sample was limited to students in grades 5 to 9, aged between 10 and 15 years. To ensure the sample size was adequate, based on the rule of thumb in social science research, the sample size should generally be 10–15 times the total number of questionnaire items (Klompstra et al., 2018). This study used three scales: the green exercise scale (5 items), the self-efficacy scale (10 items), and the kinesiophobia scale (13 items). Therefore, the minimum required sample size was between 280 and 345 participants. Data collection took place from April 28, 2025, to October 28, 2025, during which a total of 1,300 questionnaires were distributed. To ensure the validity and reliability of the data, 129 questionnaires with missing or inconsistent responses were excluded as invalid. Additionally, 72 questionnaires were identified with fixed-pattern responses during a second data check and were thus removed. The final valid sample consisted of 1,099 questionnaires, with an effective response rate of 84.54%.

### 3.2 Measurement tools

#### 3.2.1 Green exercise

This study employed the green exercise scale validated by Wood et al. (2019) to assess individuals’ participation in green exercise. The scale consists of five items designed to measure engagement in green exercise activities. Each item is rated on a 5-point Likert scale ranging from 1 (strongly disagree) to 5 (strongly agree), with higher scores indicating greater participation. The scale has undergone cross-cultural validation and has demonstrated good reliability and validity across different populations; specifically, Cronbach’s *α* typically exceeds 0.80, indicating high internal consistency. In the present study, Cronbach’s *α* was 0.891, further confirming its good reliability in our sample.

#### 3.2.2 Self-efficacy

This study used the self-efficacy scale validated by Wang et al. (2001) to evaluate individuals’ self-efficacy. The scale contains 10 items aimed at gaging overall self-efficacy. Each item is scored on a 5-point Likert scale (1 = strongly disagree, 5 = strongly agree), with higher scores reflecting stronger self-efficacy. The scale has been cross-culturally validated and has shown good reliability and validity across multiple populations; Cronbach’s *α* typically exceeds 0.80, indicating high internal consistency. In this study, Cronbach’s *α* was 0.924, further confirming the scale’s reliability in our sample.

#### 3.2.3 Kinesiophobia

This study employed the kinesiophobia scale validated by Tkachuk and Harris (2012) to assess individuals’ levels of kinesiophobia. The scale comprises two dimensions—Somatic focus and Activity avoidance—with a total of 13 items designed to capture fear experienced during physical activity. Each item is rated on a 5-point Likert scale (1 = “strongly disagree,” 5 = “strongly agree”), with higher scores indicating greater kinesiophobia. The scale has undergone cross-cultural validation and has demonstrated good reliability and validity across diverse populations, with Cronbach’s *α* typically exceeding 0.80, indicating high internal consistency. In the present study, Cronbach’s *α* for Somatic focus and Activity avoidance was 0.898 and 0.914, respectively, further confirming the scale’s reliability in our sample.

### 3.3 Data analysis

All statistical analyses were conducted using IBM SPSS Statistics 26.0 and AMOS 26.0. Prior to formal analyses, the dataset was screened for missing values, outliers, and distributional normality to verify the suitability of parametric procedures.

Descriptive statistics (means, standard deviations, and frequencies) were computed to summarize demographic characteristics and key study variables. Internal consistency for each construct—green exercise, self-efficacy, somatic focus, and activity avoidance—was evaluated using Cronbach’s *α*, with values above 0.70 considered acceptable (Ahmad et al., 2024). Composite reliability (CR) and average variance extracted (AVE) were calculated to assess construct reliability and convergent validity, using CR > 0.70, AVE > 0.50, and standardized factor loadings > 0.60 as evaluation criteria (Cheung et al., 2024).

Construct validity of the measurement model was examined via confirmatory factor analysis (CFA). In CFA, all latent constructs were indicated by their original item-level observed variables; no item parceling was used. Model fit was evaluated using multiple indices, including the chi-square to degrees of freedom ratio (*χ*^2^/df < 5), Comparative Fit Index (CFI > 0.90), Tucker–Lewis Index (TLI > 0.90), Standardized Root Mean Square Residual (SRMR < 0.08), and Root Mean Square Error of Approximation (RMSEA < 0.08) with its 90% confidence interval (CI) (Hair, 2011).

To assess potential common method bias (CMB) associated with self-report measures, Harman’s single-factor test was conducted, followed by CFA-based comparisons among alternative measurement models (one-factor, two-factor, three-factor, and the hypothesized four-factor model) (Kock et al., 2021). In addition, an Unmeasured Latent Method Construct (ULMC) model was estimated by adding a latent factor that loaded on all measurement items to control for possible method variance. CMB was considered negligible when the first unrotated factor accounted for less than 40% of the total variance and the ULMC model did not significantly improve model fit relative to the hypothesized four-factor model.

After establishing measurement adequacy, Pearson correlation analysis was performed to examine bivariate associations among the primary constructs (Tang and Wen, 2020). Multiple linear regression analyses were then conducted at the observed-score level to estimate the associations of green exercise and self-efficacy with the two kinesiophobia dimensions (somatic focus and activity avoidance), while controlling for gender, grade, and place of residence. The regression approach was used to provide covariate-adjusted estimates with interpretable variance explained (*R*^2^) and to complement the latent-variable results.

Structural equation modeling (SEM) in AMOS 26.0 was subsequently used to test the hypothesized mediation model at the latent level. Similar to CFA, SEM specified each latent construct using its original item-level indicators (no parceling). The model included direct paths from green exercise to somatic focus and activity avoidance and indirect paths via self-efficacy; global fit was evaluated using the same indices described above.

Finally, the mediating role of self-efficacy was tested using a bias-corrected percentile bootstrap with 5,000 resamples. Indirect effects were considered statistically significant when the 95% CI did not include zero (Preacher and Hayes, 2008).

## 4 Results

### 4.1 Sample characteristics

A total of 1,099 participants were included in the analysis. As shown in Table 1, among the participants, 685 (62.33%) were male and 414 (37.67%) were female. The sample covered five grade levels with a relatively balanced distribution: Grade 5 (21.75%), Grade 6 (20.20%), Grade 7 (20.02%), Grade 8 (19.65%), and Grade 9 (18.38%). With respect to place of residence, 544 (49.50%) of the participants were from rural or township areas, and 555 (50.50%) were from urban areas, indicating a nearly even distribution between rural and urban backgrounds.

**Table 1:** Demographic characteristics of the sample (*N* = 1,099).

Variable	Category	Frequency	Percentage (%)
Gender	Male	685	62.33
	Female	414	37.67
Grade	Grade 5	239	21.75
	Grade 6	222	20.20
	Grade 7	220	20.02
	Grade 8	216	19.65
	Grade 9	202	18.38
Place of residence	Rural/township	544	49.50
	Urban	555	50.50

### 4.2 Descriptive statistics and measurement reliability

Descriptive statistics, internal consistency reliability, and results of CFA for all key variables are presented in Table 2. All scales demonstrated satisfactory internal consistency reliability, with Cronbach’s *α* values ranging from 0.891 to 0.924, exceeding the conventional threshold of 0.70. The composite reliability (CR) values ranged from 0.891 to 0.925, further confirming the reliability of the constructs. The average variance extracted (AVE) values ranged between 0.552 and 0.639, meeting the recommended criterion of 0.50, thereby indicating adequate convergent validity. The standardized factor loadings (0.675–0.848) were all statistically significant and above the recommended minimum of 0.60, demonstrating that each indicator loaded strongly on its intended latent construct. Overall, the measurement model exhibited satisfactory psychometric properties and provided a solid foundation for subsequent structural analyses.

**Table 2:** Descriptive statistics, internal consistency reliability, and fit indices for CFA of key variables (*N* = 1,099).

Variable	Mean	SD	*α*	Factor loading	CR	AVE
Green exercise	3.53	0.92	0.891	0.750–0.845	0.891	0.621
Self-efficacy	3.09	0.63	0.924	0.675–0.848	0.925	0.552
Somatic focus	2.42	1.00	0.898	0.767–0.825	0.899	0.639
Activity avoidance	2.34	0.91	0.914	0.739–0.774	0.915	0.573

SD, standard deviation; α, Cronbach’s alpha; CR, composite reliability; AVE, average variance extracted; the mean was calculated using the original 1–5 Likert scale.

### 4.3 Common method bias test

To evaluate the potential influence of CMB arising from the use of self-reported questionnaires, Harman’s single-factor test was first conducted. The unrotated exploratory factor analysis extracted four factors with eigenvalues greater than 1, jointly accounting for 64.46% of the total variance. The first factor explained 35.37% of the variance, which is below the conventional threshold of 40%, suggesting that CMB is unlikely to be a serious concern in this study.

Furthermore, to provide more rigorous evidence for the absence of CMB, a series of CFAs were conducted to compare the fit indices of alternative measurement models. As presented in Table 3, the hypothesized four-factor model (comprising green exercise, self-efficacy, somatic focus, and activity avoidance) exhibited a substantially better model fit (*χ*^2^/df = 2.880, CFI = 0.950, TLI = 0.961, SRMR = 0.025, RMSEA = 0.041) than the alternative one-, two-, and three-factor models. These fit indices met conventional cutoff criteria (CFI and TLI > 0.90; RMSEA < 0.08; SRMR < 0.08), indicating that the constructs were empirically distinct.

**Table 3:** Fit indices for alternative measurement models (*N* = 1,099).

Model	*χ*^2^/d*f*	CFI	TLI	SRMR	RMSEA (90% CI)
One-factor	26.272	0.516	0.478	0.140	0.152 (0.149, 0.154)
Two-factor	18.029	0.675	0.648	0.133	0.125 (0.122, 0.127)
Three-factor	10.378	0.822	0.806	0.098	0.092 (0.090, 0.095)
Four-factor	2.880	0.965	0.961	0.025	0.041 (0.038, 0.044)
ULMC-factor	2.889	0.965	0.961	0.025	0.041 (0.038, 0.044)

ULMC, Unmeasured Latent Method Construct; CFI, Comparative Fit Index; TLI, Tucker–Lewis Index; SRMR, Standardized Root Mean Square Residual; RMSEA, Root Mean Square Error of Approximation; CI, confidence interval.

Additionally, a ULMC model was tested to further assess potential method variance. The ULMC model did not significantly improve model fit (*χ*^2^/df = 2.889, CFI = 0.965, TLI = 0.961, SRMR = 0.025, RMSEA = 0.041) compared to the four-factor model, reinforcing that CMB was minimal. Overall, both the Harman’s single-factor test and CFA-based comparison results confirmed that CMB did not threaten the validity of the findings.

### 4.4 Correlation analysis and discriminant validity

Pearson correlation analysis was conducted to examine the associations among the key variables, and the results are presented in Table 4. Green exercise showed a significant positive correlation with self-efficacy (*r* = 0.319, *p* < 0.001) and significant negative correlations with both somatic focus (*r* = −0.320, *p* < 0.001) and activity avoidance (*r* = −0.381, *p* < 0.001). Self-efficacy was also negatively correlated with somatic focus (*r* = −0.361, *p* < 0.001) and activity avoidance (*r* = −0.377, *p* < 0.001). In contrast, the two dimensions of kinesiophobia—somatic focus and activity avoidance—were positively correlated (*r* = 0.530, *p* < 0.001). These findings suggest that higher levels of green exercise and self-efficacy are associated with lower levels of kinesiophobia, consistent with the theoretical expectations.

**Table 4:** Correlations and discriminant validity among key variables (*N* = 1,099).

Variable	Green exercise	Self-efficacy	Somatic focus	Activity avoidance
Green exercise	0.788			
Self-efficacy	0.319^***^	0.743		
Somatic focus	−0.320^***^	−0.361^***^	0.799	
Activity avoidance	−0.381^***^	−0.377^***^	0.530^***^	0.757

^***^p<0.001. Values on the diagonal represent the square roots of the AVE.

To further evaluate discriminant validity, the square roots of the AVE were compared with the inter-construct correlations. As shown on the diagonal of Table 4, the square roots of AVE (ranging from 0.743 to 0.799) exceeded the corresponding inter-construct correlation coefficients, thereby meeting the Fornell-Larcker criterion. This result indicates that each construct possessed adequate discriminant validity and represented distinct latent dimensions, providing a reliable measurement foundation for subsequent structural model testing.

### 4.5 Multiple regression analysis

To further examine the associations of green exercise and self-efficacy with the two dimensions of kinesiophobia, multiple regression analyses were conducted while controlling for gender, grade, and place of residence. Table 5 reports standardized regression coefficients (*β*), which allow effect magnitudes to be compared across predictors.

**Table 5:** Multiple regression analysis (*N* = 1,099).

Variables	Model 1 (somatic focus)		Model 2 (activity avoidance)	
	*β*	SE	*β*	SE
Control variables				
Gender	−0.006	0.057	−0.048	0.051
Grade	0.118^***^	0.020	0.104^***^	0.017
Place of residence	0.058^*^	0.055	0.038	0.049
Independent variable				
Green exercise	−0.217^***^	0.032	−0.289^***^	0.028
Mediator				
Self-efficacy	−0.279^***^	0.047	−0.266^***^	0.042
Model summary				
*R* ^2^	0.194	0.232		
Adjust *R*^2^	0.191	0.228		
*F*	52.713^***^	65.993^***^		

Standardized coefficients (*β*) are reported; ^*^p < 0.05; ^***^ *p* < 0.001.

For Model 1 (somatic focus as the dependent variable), grade (*β* = 0.118, *p* < 0.001), place of residence (*β* = 0.058, *p* < 0.05), green exercise (*β* = −0.217, *p* < 0.001), and self-efficacy (*β* = −0.279, *p* < 0.001) were significant predictors. The positive coefficients for grade and residence indicate that students in higher grades and those living in urban areas tended to report slightly higher somatic focus. In contrast, higher levels of green exercise and self-efficacy were associated with lower somatic focus. The model explained 19.4% of the variance in somatic focus (*R*^2^ = 0.194; *F* = 52.713, *p* < 0.001).

For Model 2 (activity avoidance as the dependent variable), grade (*β* = 0.104, *p* < 0.001), green exercise (*β* = −0.289, *p* < 0.001), and self-efficacy (*β* = −0.266, *p* < 0.001) were significant predictors, whereas gender and place of residence were not. Greater participation in green exercise and higher self-efficacy were associated with lower activity avoidance. This model explained 23.2% of the variance in activity avoidance (*R*^2^ = 0.232; *F* = 65.993, *p* < 0.001).

Across both models, interpretation focuses on effect-size information in addition to statistical significance. Although both models reached *p* < 0.001, the standardized coefficients suggest small-to-moderate associations (|*β*| = 0.217–0.289 for green exercise and |*β*| = 0.266–0.279 for self-efficacy), and the explained variance is modest (*R*^2^ = 0.194–0.232). Overall, the regression results indicate that green exercise and self-efficacy are negative correlates of somatic focus and activity avoidance, consistent with the hypothesized pattern evaluated further in the mediation analyses.

### 4.6 Structural equation modeling analysis

To further test the hypothesized structural relationships among the study variables, SEM was conducted using the maximum likelihood estimation method. The overall model demonstrated a satisfactory fit to the data, with the following indices: *χ*^2^/df = 2.880, CFI = 0.965, TLI = 0.961, SRMR = 0.025, and RMSEA = 0.041 [90% CI (0.038, 0.044)]. All fit indices met the recommended criteria (*χ*^2^/df < 5, CFI and TLI > 0.90, SRMR < 0.08, RMSEA < 0.08), indicating that the proposed model adequately represented the observed data.

**Figure 2:**
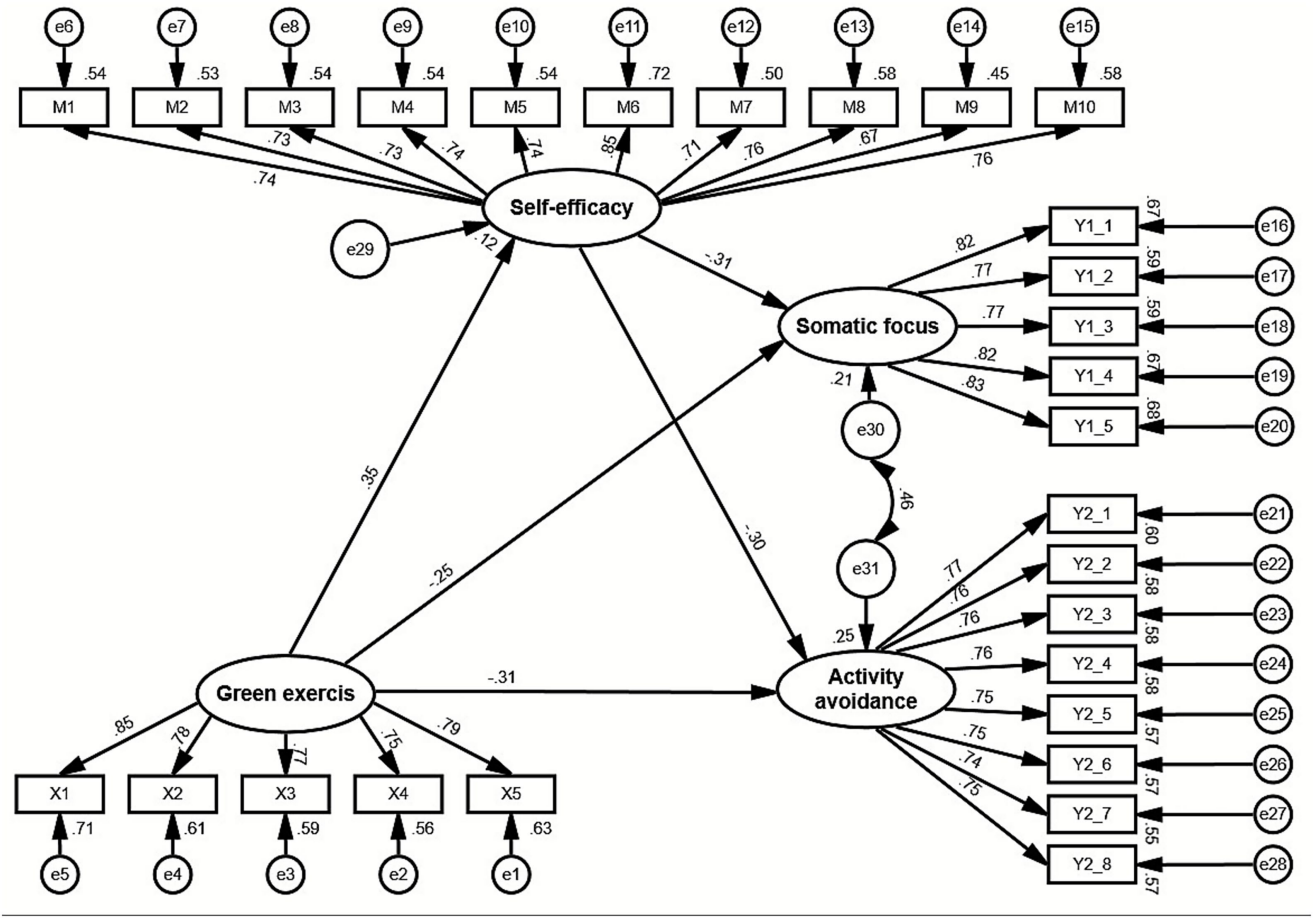
Standardized path coefficients of the structural model.

As illustrated in Figure 2, green exercise exerted a significant positive effect on self-efficacy (*β* = 0.351) and significant negative effects on both somatic focus (*β* = −0.251) and activity avoidance (*β* = −0.315). In addition, self-efficacy had significant negative effects on somatic focus (*β* = −0.308) and activity avoidance (*β* = −0.299). All path coefficients were statistically significant at *p* < 0.01. These results suggest that green exercise not only directly reduces both dimensions of kinesiophobia but also enhances self-efficacy, which in turn further decreases somatic focus and activity avoidance. Overall, the SEM results provide strong empirical support for hypotheses H1 and H2, confirming that green exercise has significant direct negative effects on the two dimensions of kinesiophobia. The subsequent mediation analyses will further test whether self-efficacy mediates these relationships.

### 4.7 Bootstrapping mediation test

To examine whether self-efficacy mediated the effects of green exercise on the two dimensions of kinesiophobia, a multiple mediation model was tested using the bias-corrected percentile Bootstrap method with 5,000 resamples. The results are presented in Table 6.

**Table 6:** Total, direct and indirect effects in the multiple mediator model (*N* = 1,099).

Dependent variable	Path	*β*	Boot SE	Boot LLCI	Boot ULCI	Ratio (%)
Somatic focus	Direct effect: green exercise → somatic focus	−0.251^***^	0.034	−0.316	−0.181	69.92
	Indirect effects: green exercise → self-efficacy → somatic focus	−0.108^***^	0.014	−0.137	−0.081	30.08
	Total effect	−0.359^***^	0.032	−0.419	−0.295	100
Activity avoidance	Direct effect: green exercise → activity avoidance	−0.315^***^	0.031	−0.376	−0.253	75.00
	Indirect effects: green exercise → self-efficacy → activity avoidance	−0.105^***^	0.014	−0.133	−0.081	25.00
	Total effect	−0.420^***^	0.028	−0.475	−0.364	100

^***^p < 0.001. Boot LLCI, the lower bound of the 95% CI. Boot ULCI, the upper limit of the 95% CI (percentile bootstrap method with bias correction). The Bootstrap sample size is set at 5000.

For somatic focus, the direct effect of green exercise on somatic focus remained significant (*β* = −0.251, *p* < 0.001), while the indirect effect through self-efficacy was also significant [*β* = −0.108, Boot SE = 0.014, 95% CI (−0.137, −0.081)]. The total effect of green exercise on somatic focus was −0.359 (*p* < 0.001), with the indirect pathway accounting for 30.08% of the total effect. These results indicate that self-efficacy partially mediates the relationship between green exercise and somatic focus.

For activity avoidance, the direct effect of green exercise was significant (*β* = −0.315, *p* < 0.001), and the indirect effect via self-efficacy was also significant [*β* = −0.105, Boot SE = 0.014, 95% CI (−0.133, −0.081)]. The total effect was −0.420 (*p* < 0.001), with 25.00% of this effect transmitted through self-efficacy. This pattern likewise indicates a partial mediating effect.

Taken together, these findings reveal that green exercise not only directly reduces both somatic focus and activity avoidance but also indirectly decreases them by enhancing self-efficacy. Therefore, the results provide empirical support for hypotheses H3 and H4, confirming that self-efficacy plays a significant mediating role in the relationship between green exercise and kinesiophobia.

## 5 Discussion

### 5.1 Overview of the findings

The results support a consistent pattern in which green exercise is associated with lower kinesiophobia among adolescents, both directly and indirectly through self-efficacy. In the regression analyses (Table 5), green exercise and self-efficacy were significant negative correlates of somatic focus (*β* = −0.217; *β* = −0.279) and activity avoidance (*β* = −0.289; *β* = −0.266), with relatively modest explained variance (*R*^2^ = 0.194 and 0.232, respectively). In the SEM mediation model (Table 6), green exercise showed significant direct associations with both dimensions of kinesiophobia, while also demonstrating significant indirect associations via self-efficacy. Bootstrap results indicated partial mediation, with indirect effects accounting for 30.08% of the total effect on somatic focus and 25.00% of the total effect on activity avoidance.

Within the framework of social cognitive theory, this mediation pattern is consistent with the proposition that efficacy beliefs shape how individuals appraise and regulate physical exertion and bodily sensations (Bandura, 1977; Bandura, 1982). Engagement in green exercise may provide experiences that strengthen perceived capability to manage exertion, as reflected in the positive path from green exercise to self-efficacy and the negative paths from self-efficacy to both kinesiophobia dimensions (Table 6). Accordingly, adolescents with higher self-efficacy tended to report less fear-related attention to bodily cues and lower activity avoidance, aligning with the convergent evidence across correlational, regression, and SEM results.

Given the cross-sectional design, these findings should be interpreted primarily as associations rather than definitive causal effects. The *R*^2^ values further suggest that green exercise and self-efficacy are meaningful yet partial contributors to kinesiophobia, which is consistent with the view that adolescent kinesiophobia is likely jointly shaped by multiple contextual, physiological, and psychosocial factors (Vlaeyen and Linton, 2000).

### 5.2 Effects of green exercise on kinesiophobia

Across all analyses, green exercise was negatively associated with both dimensions of kinesiophobia—somatic focus and activity avoidance—indicating that adolescents who reported more frequent green exercise generally showed lower fear-related attention to bodily sensations and less avoidance of physical activity. This pattern goes beyond the physiological benefits of exercise and is consistent with the view that natural environments may be linked to more adaptive emotional and cognitive responses during exertion (Bratman et al., 2015; Berman et al., 2008).

Within fear-avoidance accounts of pain and movement, threatening interpretations of bodily sensations can heighten vigilance and promote avoidance (Vlaeyen and Linton, 2000). The negative associations observed in the present study suggest that green exercise may be related to a weaker fear-avoidance cycle, insofar as activity in natural environments is often experienced as less threatening and more restorative. Natural contexts may facilitate attentional recovery and reduce emotional arousal, making exertion-related cues more tolerable and less likely to trigger avoidance motivation (Kaplan, 1995; Sudimac et al., 2022).

Green exercise may also align with corrective learning processes that weaken catastrophic beliefs about exertion. Outdoor activity can provide repeated opportunities to experience bodily sensations in a relatively benign context, supporting cognitive reappraisal and strengthening alternative interpretations of exertion as manageable rather than harmful (Jacoby and Abramowitz, 2016). Over time, such experiences may be associated with reduced salience of fear-related cues and a lower tendency to avoid movement.

Consistent with prior work positioning green exercise as an environmental context with cognitive-behavioral relevance, our findings indicate that the accessibility of, and engagement in, green exercise is associated with lower somatic focus and activity avoidance (Ramezanghorbani et al., 2025; Sprague et al., 2022). This interpretation highlights the potential psychological value of green spaces for adolescents; however, given the cross-sectional design, the results should be understood as associations rather than definitive causal effects.

### 5.3 The mediating role of self-efficacy

The results indicated that self-efficacy partially mediated the association between green exercise and kinesiophobia. In the structural model, green exercise was positively associated with self-efficacy, whereas self-efficacy was negatively associated with both somatic focus and activity avoidance. Bootstrap tests supported two significant indirect effects via self-efficacy, with the indirect pathway accounting for 30.08% of the total effect of green exercise on somatic focus and 25.00% of the total effect on activity avoidance (Table 6). Importantly, the direct paths remained significant in the presence of the indirect effects, indicating partial rather than full mediation.

This mediation pattern is consistent with social cognitive theory, which conceptualizes self-efficacy as a central mechanism influencing behavioral regulation and emotional responses under perceived risk (Bandura, 1977). Green exercise may enhance self-efficacy through experiences aligned with perceived competence during physical activity, such as repeated successful task completion and learning to manage effort-related bodily sensations in a less threatening context. Higher self-efficacy, in turn, is consistent with lower fear-based appraisal of bodily cues and reduced avoidance motivation (Benight and Bandura, 2004), which aligns with the negative associations between self-efficacy and both kinesiophobia dimensions observed in the regression and SEM results.

Partial mediation also suggests that self-efficacy is important but not the only pathway. Green exercise retained direct associations with somatic focus and activity avoidance, implying that other mechanisms may operate in parallel, such as affective restoration or attentional recovery elicited by natural environments. Overall, the findings support the view that efficacy beliefs constitute an important cognitive mechanism linking engagement in green exercise to lower kinesiophobia, while also indicating that multiple pathways may jointly shape fear-of-movement outcomes during adolescence.

### 5.4 Theoretical implications

By linking an environmental exposure context (green exercise) with a core social-cognitive mechanism (self-efficacy), this study offers a theoretical contribution to understanding kinesiophobia. Empirically, green exercise was negatively associated with both somatic focus and activity avoidance, and self-efficacy partially mediated these associations. This pattern supports an integrated interpretation in which the relationship between environmental engagement and kinesiophobia operates partly through efficacy-related appraisal processes, rather than relying on physiological mechanisms alone.

From the perspective of social cognitive theory, the findings further underscore self-efficacy as a key regulatory construct that shapes how adolescents interpret and cope with exertion-related bodily sensations under perceived risk (Bandura, 1977). The positive association between green exercise and self-efficacy, together with the negative associations between self-efficacy and both kinesiophobia dimensions, aligns with the proposition that contexts supporting perceived competence are associated with lower fear-based appraisal and reduced avoidance tendencies. In this way, the present study extends existing theoretical accounts by highlighting efficacy beliefs as a potential mechanistic bridge linking environmental contexts to movement-related fear responses.

The partial mediation results also indicate that cognitive appraisal is important but not the sole mechanism. Even when self-efficacy was included as a mediator, green exercise retained direct associations with both kinesiophobia dimensions. This is consistent with multi-process explanations in environmental psychology, which propose that nature exposure often influences mental health through pathways such as affective restoration and attentional recovery (Markevych et al., 2017; Kuo, 2015). Integrating these perspectives, our findings support a framework in which environmental affordances may relate to fear-related outcomes through both efficacy-based appraisal processes and other complementary pathways.

Overall, these theoretical implications suggest that kinesiophobia reflects an interplay between person-level cognitions and contextual features of activity settings. The findings emphasize the value of understanding efficacy beliefs and avoidance responses within the environments in which adolescents engage in physical activity, thereby providing a theoretical basis for future research connecting ecological exposure with self-regulatory cognition.

### 5.5 Practical implications

The findings have practical relevance for school-based health promotion because green exercise and self-efficacy showed small-to-moderate associations with both kinesiophobia dimensions, while the regression models explained 19–23% of the variance. This magnitude suggests that green exercise is unlikely to address kinesiophobia on its own, but it may contribute meaningfully as one component within a broader approach to adolescent physical activity and health education.

In Chinese middle-school settings, feasible implementation can be embedded within routine physical education. Examples include incorporating outdoor walking or running segments, green-space-based circuit activities, and nature-oriented games into PE classes, thereby increasing exposure to natural environments without requiring major curricular changes. Because self-efficacy partially mediated the green exercise–kinesiophobia association, outdoor activities may be more effective when paired with mastery-supportive teaching practices that strengthen perceived competence, such as graded task difficulty, clear skill feedback, achievable performance goals, and opportunities for repeated successful participation. Such design aligns with the observed role of self-efficacy and may be particularly relevant for students who are prone to fear-based avoidance.

The results also imply that efforts to increase green exercise opportunities should be framed in realistic terms consistent with the observed effect sizes. Even modest increases in outdoor physical activity time or frequency may be associated with measurable differences in fear-related outcomes, especially when implemented consistently and combined with supports that build confidence during exertion. At the school-management level, practical steps include improving access to usable green spaces on or near campus, scheduling regular outdoor activity sessions, and ensuring safe, supervised environments that facilitate participation.

At the community level, the findings are consistent with the value of accessible green infrastructure for adolescent well-being. Collaboration among schools, local communities, and urban planning stakeholders to maintain nearby parks or green corridors may expand opportunities for routine outdoor activity. Interpreted within the limits of a cross-sectional design, the present evidence supports the practical consideration of green exercise as a scalable, low-cost supplement to existing physical activity promotion efforts, with potential benefits partly linked to strengthening self-efficacy.

### 5.6 Limitations and future directions

Despite the robustness of the findings and the rigor of the analytical procedures, several methodological and conceptual limitations should be acknowledged to guide future research.

First, the cross-sectional design precludes definitive causal inference regarding the directional relationships among green exercise, self-efficacy, and kinesiophobia. Although the structural model was theoretically grounded and statistically supported, longitudinal or experimental designs are needed to determine whether engagement in green exercise produces sustained changes in self-efficacy and fear-related behavior over time. Prospective or intervention-based studies would enable the examination of temporal dynamics and causal mechanisms more directly.

Second, all data were collected through self-report measures, which may be subject to response bias and shared method variance despite the diagnostic checks indicating minimal CMB. Incorporating objective behavioral indicators, physiological measures, or ecological momentary assessments could provide convergent validity and reduce the potential influence of social desirability and retrospective recall errors. Future research should also consider multi-informant or multimodal approaches to capture the interplay between subjective experience and observable behavioral change.

Third, the sample was composed exclusively of school-aged participants from a specific sociocultural context (students from three provinces in China). While this homogeneity enhances internal validity, it constrains the generalizability of the results to other countries, age groups, or populations with clinical or chronic pain conditions. Comparative research across developmental stages, cultural backgrounds, and physical health statuses would allow the identification of boundary conditions for the green exercise–self-efficacy–kinesiophobia mechanism. Additionally, examining potential gender or contextual moderators may reveal differential sensitivity to environmental or cognitive interventions.

Finally, although the model successfully integrated environmental and cognitive determinants, it did not account for potential affective mediators such as stress reduction, mood enhancement, or attentional restoration, which may operate in parallel with self-efficacy. Incorporating these factors into a multilevel structural model could enrich theoretical understanding of how environmental experiences translate into psychological resilience. Future studies should adopt a multi-process perspective, situating self-efficacy within a broader network of emotional and cognitive pathways that connect environmental exposure to health-related behavior.

Overall, these limitations outline clear trajectories for future inquiry. Advancing this line of research requires combining experimental manipulation, ecological measurement, and theoretical refinement to establish a comprehensive model of how natural environments shape cognitive–emotional regulation and reduce fear of movement. Such integrative work will not only strengthen the empirical foundation of green exercise research but also contribute to the broader agenda of ecological psychology and health behavior science.

## 6 Conclusion

This study demonstrates the negative association between green exercise and kinesiophobia among adolescents. It shows that engaging with natural environments not only directly reduces fear-related cognition and avoidance behaviors but also indirectly diminishes these outcomes through the enhancement of self-efficacy. Self-efficacy was found to mediate this relationship, serving as a crucial intermediary between ecological experiences and emotional and behavioral regulation. These findings emphasize the importance of integrating green exercise into adolescent health promotion programs, as it helps build confidence, reduce avoidance, and encourage sustained participation in physical activity. Overall, the results suggest that fostering interaction with natural environments is a psychologically transformative process that enhances resilience and mental health.

## Data Availability

The original contributions presented in the study are included in the article/Supplementary material, further inquiries can be directed to the corresponding author.
